# Impact of the COVID‐19 lockdown on gifted and non‐gifted primary school students' well‐being and motivation from a self‐determination perspective

**DOI:** 10.1111/1471-3802.12583

**Published:** 2022-11-01

**Authors:** H. Elise Samsen‐Bronsveld, Sanne H.G. Van der Ven, Paula P.A.M. Speetjens, Anouke W.E.A. Bakx

**Affiliations:** ^1^ Stichting BOOM The Netherlands; ^2^ Tilburg University The Netherlands; ^3^ Radboud University The Netherlands; ^4^ Netherlands Youth Institute The Netherlands; ^5^ Stichting Families Foundation The Netherlands; ^6^ Fontys Child and Education University of Applied Sciences The Netherlands; ^7^ PPF Centrum Voor Hoog OntwikkelingsPotentieel The Netherlands

**Keywords:** Lockdown, distance learning, self‐determination theory, giftedness, primary education

## Abstract

This study examined the impact of the COVID‐19‐induced school lockdown on need satisfaction, well‐being and motivation in both gifted and non‐gifted primary school students in the Netherlands. A total of 312 parents (122 from gifted children) participated. The lockdown had mainly negative effects on students' need satisfaction, well‐being and motivation. However, the impact of the lockdown was less negative for gifted students. There was also a levelling effect: Before the lockdown, gifted students had lower need satisfaction, well‐being and motivation than their non‐gifted peers, but these differences decreased during the lockdown due to (stronger) declines in the non‐gifted. Changes in non‐gifted students' well‐being and motivation, because of the lockdown, were negatively mediated by autonomy and relatedness with classmates. Among the gifted, this was positively mediated by competence. Only before the lockdown, the effects of giftedness on well‐being and motivation were mediated by autonomy and relatedness satisfaction.

## Introduction

Due to COVID‐19, schools were closed in many countries worldwide, affecting more than 1 billion learners (UNESCO, 2021). Also in the Netherlands, primary schools went into lockdown for 8 weeks. Most students were therefore confronted with distance learning. Although this had some positive effects for (some) students, for example more contact with their parents (Pozas, Letzel and Schneider, 2021), most effects were negative on academic (Engzell, Frey and Verhagen, 2020; Tomasik, Helbling and Moser, 2020), social–emotional (Christner et al., 2021) and motivational levels (Zaccoletti et al.,  2020). We investigated whether such a decrease in well‐being and motivation could be explained from a self‐determination perspective (Deci and Ryan, 1985; Ryan and Deci, 2017), which states that well‐being and motivation arise when the needs autonomy, competence and relatedness are met.

Most COVID‐19 studies investigated the effects of the lockdown on students in general. However, little is known about possible effects on gifted students, who need special educational adaptions to flourish, such as curriculum compacting, enrichment or special pull‐out programmes (for a review, see Reis and Renzulli, 2010). Even though many gifted students attend school together with non‐gifted students, or received the same distance education, their giftedness can influence their perceived autonomy, competence and relatedness.

Our study investigated whether the lockdown affected need satisfaction, well‐being and motivation of gifted and non‐gifted primary school students, according to their parents. These insights can contribute to improving education tailored to all students.

### Impact of the lockdown on primary school students

To investigate the impact of the lockdown, we used the self‐determination theory (SDT) by Deci and Ryan (1985) as a lens. They posit that everyone has three basic psychological needs: autonomy (acting according to your own will), competence (feeling capable and challenged in your abilities) and relatedness (feeling connected; Stroet, Opdenakker and Minnaert, 2013). According to the SDT, satisfaction of these needs leads to psychological growth, well‐being and motivation (Deci and Ryan, 2000; Ryan and Deci, 2001, 2017). Several studies showed that need satisfaction indeed predicted improved well‐being and motivation (Church et al., 2013; Earl et al., 2019; Wang et al., 2019).

Students' need satisfaction is influenced by their learning environment (Ryan and Deci, 2020). Teachers can stimulate their students' need for autonomy, competence and relatedness through, respectively, autonomy support (e.g., fostering choice), structure (e.g., offering guidance) and involvement (e.g., showing affection; Stroet et al., 2013). This need‐supportive teaching fosters students' well‐being and motivation. However, due to the pandemic, for many learners around the world, their learning environment changed from school to home, for a few weeks or months (e.g., in European countries) or even more than 10 months (e.g., in many schools in the USA; UNESCO, 2021). Because of this unique situation, several ad‐hoc and natural experiment‐like studies were conducted worldwide on academic, social–emotional and motivational effects of distance learning on students.

#### Academic effects

A negative impact of the lockdown was seen on the faltering academic progress of students (Engzell et al., 2020; Tomasik et al., 2020). For example, Engzell et al. (2020) showed that in an 8‐week lockdown, students had a learning delay of 6–8 weeks in maths, spelling and reading.

#### Social–emotional effects

School closure had mainly negative effects on students' social–emotional functioning and general well‐being. They had more emotional, conduct and hyperactivity problems (Cellini et al., 2021; Champeaux et al., 2020; Christner et al., 2021). On the contrary, some COVID‐19 studies indicated positive effects on well‐being. For example, pre‐existing mental health problems in students decreased because of better family functioning during the lockdown (Penner, Ortiz and Sharp, 2021). Furthermore, involvement of fathers because of the lockdown led to improved well‐being (Mangiavacchi, Piccoli and Pieroni, 2021), although this involvement had unfortunately decreased during a second lockdown (Yerkes et al., 2021).

#### Motivational effects

For some students, the lockdown negatively affected their motivation. Zaccoletti et al. (2020) found a decrease in students' academic motivation as reported by their parents. These authors suggested that this decrease could be explained by less satisfaction of autonomy, competence and relatedness. Indeed, online learning environments that are less autonomy‐supportive lead to less engaged students (Chiu, 2021a, b). Although students had less supervision from their teacher and were expected to organize and complete their tasks more independently (Chiu, 2021b), their autonomy satisfaction may have decreased. During the lockdown, parents gave their children less autonomy support (Bülow et al., 2021) and some children experienced more parental control than before the lockdown (Stoecklin et al., 2021). Furthermore, students felt less competent during the lockdown, for example, because of unclear online instructions (Chiu, 2021b). Many parents indicated that distance learning had low quality and schools offered poor support (Thorell et al., 2020), which may have reduced competence satisfaction. Finally, although many children and parents spent more time together due to the lockdown, students had fewer social contacts with their classmates, teachers and friends (Bülow et al., 2021; Pozas et al., 2021), which may have led to feelings of isolation (Thorell et al., 2020) and a lower relatedness satisfaction.

### Same impact for all?

The effects of the lockdown were not equally strong for all students. For example, interindividual differences in learning increased as a result of the lockdown (Tomasik et al., 2020). During the lockdown social–emotional difficulties increased, but these were age‐related: 7‐ to 10‐year‐olds increased more in emotional problems but less in conduct symptoms and hyperactivity than 3‐ to 6‐year‐olds (Christner et al., 2021). Furthermore, although well‐being and motivation generally decreased, this decrease was not observed in all students (Poulain et al., 2021; The Children's Society, 2020).

In the aforementioned studies, no subgroups of learners were distinguished. These are general findings that might differ for groups with specific learning characteristics. Gifted students are such a group that has certain characteristics, such as a high cognitive potential and creativity (Gagné, 2010; Pfeiffer et al., 2018), which may affect their susceptibility to the lockdown. A parallel study of our study investigated the relationship between need satisfaction, well‐being and motivation in secondary school students from a parental perspective (Hornstra et al., 2022). Findings showed that the impact of the lockdown was less negative for students with special educational needs (SEN), including gifted students. In general, before the lockdown, students with SEN scored lower on need satisfaction, well‐being and motivation than students without SEN. These differences became smaller during the lockdown or even disappeared due to a decline in need satisfaction of students without SEN.

### SDT and giftedness

#### Need satisfaction in gifted students

Both gifted and non‐gifted students need autonomy, competence and relatedness. In the study of Bakx et al. (2019), students were asked what a good teacher is for them. Gifted and non‐gifted students both reported aspects associated with relatedness, followed by competence and autonomy. Hornstra et al. (2020) found that gifted students differed from non‐gifted students in the degree of perceived satisfaction of some needs. Although gifted students reported similar levels of autonomy and relatedness with teacher satisfaction, they experienced more competence satisfaction but less relatedness with classmates’ satisfaction. Gifted students may feel less related to their classmates because they feel their differentness, for example because they learn faster or have different interests (Coleman, Micko and Cross, 2015).

#### Well‐being in gifted students

In the literature, there are two perspectives on how giftedness affects students' well‐being (Neihart, 1999). The first is that the nature of giftedness contributes to resilience and well‐being and the second is that giftedness increases vulnerability, for example because gifted students have a higher risk of adjustment problems. Several studies showed a positive relationship between giftedness and well‐being (Chehrehbarghi and Narimani, 2017; Weyns, Colpin and Verschueren, 2021), but other studies found a negative (Casino‐García, García‐Pérez and Llinares‐Insa, 2019; Eren et al., 2018) or no relationship (Bergold, Wirthwein and Steinmayr, 2020; for reviews see Jones, 2013 and Neihart, 1999). An explanation might be that well‐being is defined and measured in different ways and contexts. The present study focused on school‐related well‐being. An important condition for gifted students' well‐being at school is that their educational environment is adapted to their needs (Kroesbergen et al., 2016), but regular teaching is often not adjusted (Fraser‐Seeto, Howard and Woodcock, 2015).

#### Motivation in gifted students

Hornstra et al. (2020) investigated the relationship between gifted and non‐gifted students' need satisfaction and motivation. They showed that need satisfaction is related to improved motivation in both gifted and non‐gifted students. Furthermore, there is evidence not only that gifted students have a higher quality of motivation than non‐gifted students (Bergold et al., 2020; Hornstra et al., 2020), but also that gifted students experience more amotivation (Hornstra et al., 2020). Teachers and parents often see a lack of motivation in gifted students (Rubenstein et al., 2012). The quality of motivation depends on several factors; for example, high‐achieving gifted students are more motivated than underachieving gifted students (McCoach and Siegle, 2003). Motivation can also seem high because high achievers are often identified as gifted, whereas underachievers are not. The motivation quality also depends on the learning environment; for example, tailored education and teacher support stimulate gifted students' motivation (McCoach and Flake, 2018).

### The present study

Our study is part of a larger project into the impact of the lockdown on Dutch primary and secondary school students. The aforementioned study by Hornstra et al. (2022) was conducted with parents of secondary school students, and to our knowledge, a similar study in specifically (gifted) primary school students has not been reported before. The aim of the present study was therefore to gain a deeper understanding of the effects of the lockdown on the relation between need satisfaction on the one hand and well‐being and motivation on the other hand for primary school students in general and gifted students in particular. The research questions and hypotheses were:

How did the degree of need satisfaction, well‐being and motivation of primary school students in general and gifted students in particular change because of the lockdown?

We hypothesized that students' autonomy, competence and relatedness satisfaction decreased due to the lockdown, as students received less clear instructions and teacher support (Chiu, 2021b; Thorell et al., 2020), and all contact with their classmates and teacher was online. However, we expected that competence satisfaction decreased less in the gifted students because of their high capacities. Since previous research showed that for gifted students relatedness with classmates was lower before the lockdown (Hornstra et al., 2020), we expected that the forced isolation from classmates during the lockdown had less impact on gifted students, as for them, the difference between before and during the lockdown was smaller. We therefore expected a smaller decline in relatedness satisfaction for gifted students. Finally, we hypothesized that well‐being and motivation declined for all students.

To what extent does a change in need satisfaction, because of the lockdown, predict changes in well‐being and motivation and to what extent is this different for gifted students?

Hornstra al. (2022) found that the lockdown had a less negative impact on secondary school students with SEN, including gifted students, than on students without SEN. We therefore hypothesized that well‐being and motivation declined in all students due to a decrease in need satisfaction, but especially in the non‐gifted students, due to a stronger decline in their need satisfaction.

To what extent do gifted students and non‐gifted students differ in their well‐being and motivation, both before and during the lockdown, and are these differences mediated by need satisfaction?

We expected that gifted students had lower well‐being and motivation than non‐gifted students, especially before the lockdown, because regular education is often not adjusted to their needs (Fraser‐Seeto et al., 2015). Since online education was insufficiently tailored to both groups, we expected these differences between the two groups were smaller during the lockdown. We hypothesized that gifted students' lower well‐being and motivation were mediated by lower need satisfaction because, according to the SDT, lower need satisfaction leads to lower well‐being and motivation (Ryan and Deci, 2017). We expected this mediation effect especially before the lockdown, because the differences between the gifted and non‐gifted in their well‐being and motivation, and also in their need satisfaction, were expected to be greater before than during the lockdown.

## Method

### Procedure and respondents

The research design of the present study was approved by the ethics committee of Fontys University. The link to the questionnaire was shared online via the professional network of the research team. During the lockdown in the Netherlands (March 16–June 8, 2020), the questionnaire was available online for 3 weeks for parents of primary school students. For this study, we used parental reports. This allowed the inclusion of parents of students of all ages in primary education, including students who are not yet capable to read. Parents were informed about the study Hornstra and were able to consent to the use of their provided data. Parents completed the questionnaire about one of their primary school children at their chosen time and place.

The sample consisted of 312 parents. Table 1 presents the demographic data of these parents and their (non‐)gifted children. The children's ages ranged from 4 to 12 years old. All primary school grades were represented in the sample: 14.6% were in kindergarten, 14.2% in grade 1, 13.9% in grade 2, 15.5% in grade 3, 14.9% in grade 4, 13.9% in grade 5 and 12.9% in grade 6.

**Table 1:** Age and gender of the gifted and non‐gifted students and the educational attainment of parents who participated in the present study

	Gifted students (n = 122)	Non‐gifted students (n = 190)	Total (n = 312)
Age (*M, SD*)	8.22, 2.12	8.14, 2.24	8.17, 2.19
Gender (%)			
Boys	63.9	51.1	56.1
Girls	36.1	48.9	43.9
Educational attainment of parents (%)			
Academic education	52.5	38.4	43.9
Professional education	43.4	45.8	44.9
Vocational education	4.1	14.2	10.3
Primary and/or secondary education	0.0	1.5	0.9

In the total sample, 39.1% of the parents indicated that their child had characteristics of giftedness. Although there is no unambiguous definition of giftedness, mainly an above‐average ability (IQ ≥120 or very high school achievements), but also creativity and task commitment are generally seen as characteristics of giftedness in the Dutch school context. Many European countries, also the Netherlands, use intelligence tests but for education, more often teacher and parent nominations are used to identify giftedness (Sękowski and Łubianka, 2015). For the present study, parents were therefore asked how the giftedness of their child was identified. In most cases, it was identified with an intelligence test (60.6%: 47.1% IQ ≥130 and 13.5% IQ 120–130). In the other cases, the giftedness was identified by both school and parents (21.4%), only school (9.0%) and only parents (9.0%). The latter is not always a reliable source. Therefore, we conducted all analyses with and without the 11 students nominated by their parents only. We reported the full results: The only difference found has been reported in the results. The remaining parents (60.9%) indicated that their child had no characteristics of giftedness.

### Instruments

We used the same instruments as the parallel study of Hornstra et al. (2022). Online questionnaires were administered to investigate need satisfaction, well‐being and motivation. All items were adjusted to create a parental version and adapted to the context of the present study – the global pandemic. We used a retrospective pretest–posttest design (Little et al., 2020). Each question was therefore asked twice: once in retrospect about the situation before the lockdown (education at school) and once about the situation during the lockdown (online education).

#### Need satisfaction

To investigate to what extent students' autonomy, competence and relatedness with classmates were met, we used the Basic Needs Satisfaction and Frustration Scale (Chen et al., 2015) and relatedness with teacher was assessed with the questionnaire of Peetsma, Wagenaar and De Kat (2001). A 5‐point Likert scale was used, ranging from 1 (*totally disagree*) to 5 (*totally agree*).

##### Autonomy

Autonomy was measured with the 8‐item subscale ‘autonomy satisfaction’. An example is: School situation [or: At home/online education]: My child felt [or: feels] that he/she had [or: has] a sense of choice or freedom in the things he/she did [or: does]. The internal consistency was good before (α = 0.82) and during (α = 0.80) the lockdown.

##### Competence

Competence was assessed using the 3‐item subscale ‘competence satisfaction’. An example is: School situation [or: At home/online education]: My child felt [or: feels] that he/she can successfully complete difficult tasks. The competence subscale had a good internal consistency before the lockdown (α = 0.82) and an acceptable internal consistency during the lockdown (α = 0.76).

##### Relatedness with classmates

Relatedness with classmates was measured with a 4‐item subscale. An example is: School situation [or: At home/online education]: My child was [or: is] involved with classmates [even though we are at home]. The first two items were removed, because the internal consistency was unacceptable, both of the subscale before the lockdown (α = 0.43) and of the subscale during the lockdown (α = 0.30). After removing these items, the internal consistency of both scales was acceptable (resp. α = 0.68; α = 0.71).

##### Relatedness with teacher

Relatedness with teacher was measured with an adapted 6‐item version of the scale ‘Well‐being with the teacher’ (Peetsma et al., 2001). An example is: School situation [or: At home/online education]: When my child felt [or: feels] unhappy, he/she can talk about it with the mentor/teacher/coach. The internal consistency of the relatedness with teacher subscale was very good before the lockdown (α = 0.92) and good during the lockdown (α = 0.89).

#### Well‐being and motivation

Students' well‐being was measured by two items: two for the situation before the lockdown (How did your child feel at school while the schools were open?; How was your child doing while the schools were still open?) and two parallel items for during the lockdown (How does your child feel now, in the situation of homeschooling?; How is your child doing now?). The internal consistency of the well‐being scale was good before (α = 0.89) and during (α = 0.84) the lockdown. A 5‐point scale was used, ranging from 1 (*not good at all*) to 5 (*very good*). Motivation was measured by two parallel items: one for the situation before the lockdown (Was your child motivated for schoolwork?) and one for the situation during the lockdown (Is your child motivated for schoolwork in the new situation?). Again, a 5‐point scale was used, ranging from 1 (*not motivated at all*) to 5 (*very motivated*).

### Analyses

The data were analysed using IBM SPSS Statistics 27. First, mixed ANOVAs with lockdown (before/during lockdown) as a within‐subjects factor, giftedness (gifted/non‐gifted) as a between‐subjects factor and the interaction of lockdown * giftedness were conducted to investigate the effects of the lockdown on need satisfaction, well‐being and motivation in (gifted) students. If the interaction effect was significant, two one‐way ANOVAs were conducted, separately before and during the lockdown.

Second, Model 1 of the MEMORE tool (Montoya and Hayes, 2017) was used to investigate to what extent the effects of the lockdown on well‐being and motivation were mediated by changes in need satisfaction. This tool creates difference scores for the measurements: during – before the lockdown. To compare the differences between the non‐gifted and the gifted group, the file was split and unstandardized variables were used. Four mediation models were created: two for the gifted and two for the non‐gifted students. Lockdown (a dummy variable) was the independent variable, well‐being (during – before the lockdown) and motivation (during – before the lockdown) were the respective dependent variables, and the needs (during – before the lockdown) were included as mediator variables. The unstandardized coefficients (*B*) and the 95% confidence interval (CI) are reported.

Thereafter, Model 4 of the PROCESS tool (Hayes, 2017) was used to examine to what extent differences between gifted and non‐gifted students in their well‐being and motivation were mediated by need satisfaction. All continuous variables were standardized for this model. Four mediation models were created: two for before and two for during the lockdown. Giftedness was the independent variable, well‐being and motivation were the respective dependent variables, and the needs were parallel mediators. Bootstrapping with 5000 samples was used. The unstandardized coefficients (*B*) and the 95% confidence interval (CI) are reported.

For all analyses, we checked the assumptions. Histograms and q‐q plots showed that the residuals of all dependent variables were normally distributed. The assumptions of homogeneity (ANOVAs), homoscedasticity and linearity (scatterplots with standardized predicted values and residuals) were also met. We found one outlier on relatedness with teacher before the lockdown. Our analyses were conducted with and without this outlier: The outlier did not lead to different conclusions, so we report the full results.

## Results

### Descriptive statistics

All measures of well‐being, motivation and need satisfaction presented here are according to the students' parents. For readability purposes, we leave out ‘according to parents’. Table 2 shows the descriptive statistics of the total sample and the two subgroups.

**Table 2:** Means (*M*) and standard deviations (*SD*) of need satisfaction, well‐being and motivation of all students before the lockdown and during the lockdown

	Gifted students (n = 122)		Non‐gifted students (n = 190)		Total (n = 312)	
	*M*	*SD*	*M*	*SD*	*M*	*SD*
Autonomy						
Before the lockdown	2.62	0.74	3.01	0.54	2.86	0.65
During the lockdown	2.72	0.77	2.82	0.67	2.78	0.71
Competence						
Before the lockdown	3.59	0.97	3.69	0.75	3.65	0.84
During the lockdown	3.81	0.82	3.69	0.84	3.74	0.83
Relatedness with classmates						
Before the lockdown	3.66	0.84	3.87	0.76	3.79	0.80
During the lockdown	2.60	0.98	2.76	1.01	2.70	1.00
Relatedness with the teacher						
Before the lockdown	3.58	0.90	3.87	0.64	3.76	0.77
During the lockdown	3.06	0.95	3.02	0.85	3.03	0.89
Well‐being						
Before the lockdown	3.57	1.04	4.07	0.68	3.88	0.85
During the lockdown	3.64	0.88	3.82	0.73	3.75	0.79
Motivation						
Before the lockdown	3.39	1.22	3.84	0.87	3.66	1.04
During the lockdown	3.17	0.98	3.32	0.92	3.26	0.94

The correlations between the variables of the present study before and during the lockdown are presented in Table 3. Almost all correlations were significant, except (1) competence and relatedness with classmates during the lockdown and (2) the autocorrelation of well‐being before and during the lockdown. Before the lockdown, giftedness was negatively correlated with satisfaction of all needs (except competence), well‐being and motivation. During the lockdown, giftedness was no longer correlated with these factors (except well‐being).

**Table 3:** Correlation matrix of the model variables need satisfaction, well‐being and motivation before and during the lockdown

		During the lockdown						
		1	2	3	4	5	6	7^a^
Before the lockdown	1. Autonomy	0.22**	0.50**	0.26**	0.31**	0.50**	0.58**	−0.07
	2. Competence	0.58**	0.33**	0.06	0.12*	0.52**	0.51**	0.07
	3. Relatedness with classmates	0.38**	0.37**	0.21**	0.42**	0.18**	0.29**	−0.08
	4. Relatedness with the teacher	0.62**	0.55**	0.49**	0.39**	0.17**	0.21**	0.02
	5. Well‐being	0.65**	0.64**	0.51**	0.60**	−0.08	0.54**	−0.11*
	6. Motivation	0.62**	0.60**	0.40**	0.50**	0.66**	0.15**	−0.08
	7. Giftedness^a^	−0.30**	−0.06	−0.13*	−0.19**	−0.28**	−0.21**	–

*Notes*: Below the diagonal are the correlations between the variables before the lockdown and above the diagonal are the correlations between the variables during the lockdown. On the diagonal, autocorrelations are shown between the same constructs before and during the lockdown. Giftedness was the same before and during the lockdown.

^a^ 0 = non‐gifted, 1 = gifted.

*P < 0.05, **P < 0.01, ***P < 0.001.

### Impact of the lockdown on gifted and non‐gifted students' need satisfaction, well‐being and motivation

To investigate how the lockdown affected need satisfaction, well‐being and motivation in gifted and non‐gifted children, a series of mixed ANOVAs were conducted (Table 4). One‐way ANOVAs were conducted if there was an interaction effect of lockdown * giftedness, separately before and during the lockdown (Table 5).

**Table 4:** Mixed ANOVAs: impact of the lockdown and giftedness on need satisfaction, well‐being and motivation in primary school students

	Sum of squares	Mean square	*F* (1, 310)	*P*	η_p_^2^
Autonomy					
Lockdown	0.33	0.33	0.93	0.337	0.003
Giftedness	9.10	9.10	17.00	**<** **0** **.001**	0.052*
Lockdown * Giftedness	3.18	3.18	9.04	**0** **.003**	0.028*
Competence					
Lockdown	1.72	1.72	3.67	0.056	0.012
Giftedness	0.01	0.01	0.01	0.945	0.000
Lockdown * Giftedness^a^	1.83	1.83	3.91	0.049	0.012*
Relatedness with classmates					
Lockdown	174.40	174.40	265.88	**<0.001**	0.462***
Giftedness	4.95	4.95	5.12	**0.024**	0.016*
Lockdown * Giftedness	0.07	0.07	0.11	0.742	0.000
Relatedness with teacher					
Lockdown	70.63	70.63	173.63	**<0.001**	0.355***
Giftedness	2.30	2.30	2.40	0.122	0.008
Lockdown * Giftedness	4.17	4.17	10.09	**0.002**	0.032*
Well‐being					
Lockdown	1.34	1.34	1.79	0.182	0.006
Giftedness	17.54	17.54	30.01	**<0.001**	0.088**
Lockdown * Giftedness	3.82	3.82	5.11	**0.024**	0.016*
Motivation					
Lockdown	19.74	19.74	23.81	**<0.001**	0.071**
Giftedness	13.40	13.40	12.19	**0.001**	0.038*
Lockdown * Giftedness	3.40	3.40	4.11	**0.044**	0.013*

*Notes*: Bold results are significant; *Small effect; **Moderate effect; ***Large effect.

^a^ The interaction effect lockdown * giftedness on competence was no longer significant if the 11 gifted students nominated by their parents only were excluded, *F*(1, 299) = 3.09, *P* = 0.060.

**Table 5:** One‐way ANOVAs: differences between gifted and non‐gifted students' need satisfaction, well‐being and motivation before and during the lockdown

	Sum of squares	Mean square	*F* (1, 310)	*P*	η_p_^2^
Autonomy					
Before the lockdown	11.52	11.52	29.66	**<0.001**	0.087**
During the lockdown	0.76	0.76	1.52	0.218	0.005
Competence					
Before the lockdown	0.83	0.83	1.17	0.218	0.004
During the lockdown	1.01	1.01	1.47	0.228	0.005
Relatedness with teacher					
Before the lockdown	6.33	6.33	11.07	**0.001**	0.034*
During the lockdown	0.14	0.14	0.18	0.676	0.001
Well‐being					
Before the lockdown	18.87	18.87	26.70	**<0.001**	0.079**
During the lockdown	2.49	2.49	3.98	**0.047**	0.013*
Motivation					
Before the lockdown	15.15	15.15	14.55	**<0.001**	0.045*
During the lockdown	1.65	1.65	1.86	0.174	0.006

*Notes*: Bold results are significant. *Moderate effect. **Small effect.

**Figure 1:**
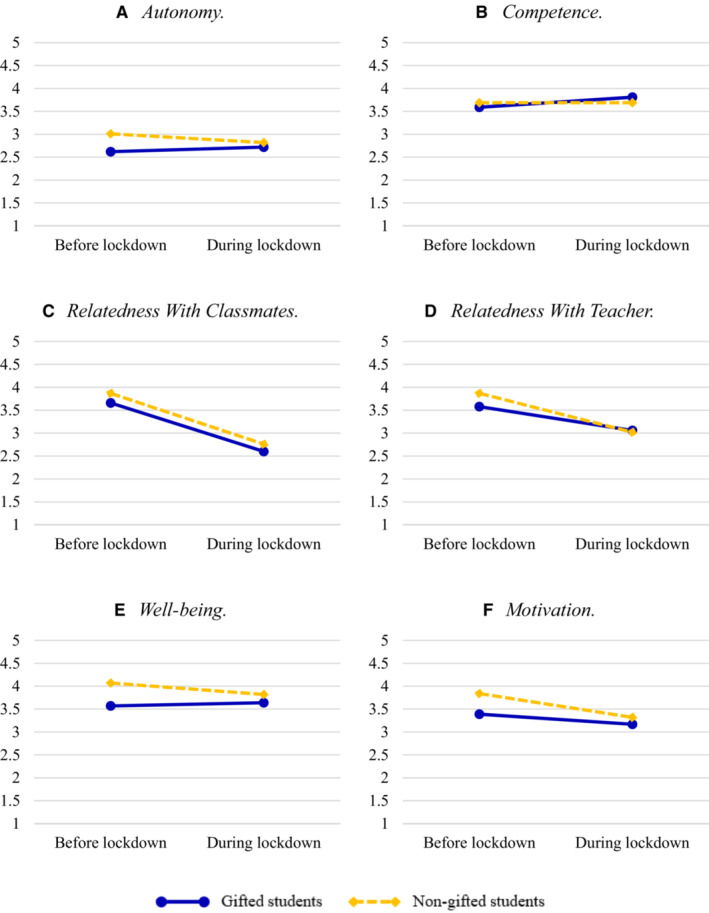
Means of satisfaction of the basic psychological needs, well‐being and motivation of gifted and non‐gifted students

The mixed ANOVA with autonomy as dependent variable showed no significant main effect of the lockdown. The main effect of giftedness was small but significant: Gifted students had lower autonomy satisfaction than non‐gifted students. The results showed a small but significant interaction effect of lockdown * giftedness, which is illustrated in Figure 1a. Two one‐way ANOVAs were conducted to interpret this interaction effect. Before the lockdown, gifted students scored significantly lower on autonomy satisfaction compared with the non‐gifted students, a moderate effect. During the lockdown, gifted students did not differ significantly from the non‐gifted students.

The mixed ANOVA on competence indicated no significant main effect of the lockdown or giftedness. The interaction effect of lockdown * giftedness was small but significant. As shown in Figure 1b, before the lockdown satisfaction of competence was slightly lower for gifted than for non‐gifted students, but in gifted students, it increased more strongly than in non‐gifted students. Group differences remained so small, however, that two one‐way ANOVAs showed no significant differences between gifted and non‐gifted students, neither before the lockdown nor during the lockdown.

The mixed ANOVA on relatedness with classmates (Figure 1c) revealed a large significant main effect of the lockdown. On average, students' relatedness with classmates’ satisfaction was markedly lower during the lockdown. The main effect of giftedness was small but significant: Gifted students showed less satisfaction than non‐gifted students. The interaction effect of lockdown * giftedness was not significant.

The mixed ANOVA on relatedness with teacher showed a large significant main effect of the lockdown. On average, students had lower relatedness with teacher satisfaction during the lockdown than before. The main effect of giftedness was not significant. The interaction effect of lockdown * giftedness was small but significant, see Figure 1d. One‐way ANOVAs indicated that before the lockdown, gifted students had lower relatedness with teacher satisfaction than non‐gifted students (a small effect) but during the lockdown and there were no significant differences.

The mixed ANOVA on well‐being showed no significant main effect of the lockdown. The main effect of giftedness was moderately sized significant: Gifted students had lower well‐being than the non‐gifted. The interaction effect of lockdown * giftedness was small but significant, see Figure 1e. Two one‐way ANOVAs showed that both before and during the lockdown, gifted students scored significantly lower on well‐being than non‐gifted students. However, before the lockdown, this was a moderate effect and after the lockdown this effect was small.

The mixed ANOVA on motivation revealed a moderately sized significant main effect of the lockdown. On average, students' motivation was lower during the lockdown than before. The main effect of giftedness was small but significant: Gifted students were less motivated than the non‐gifted. The interaction effect of lockdown * giftedness was small but significant, see Figure 1f. Two one‐way ANOVAs showed that before the lockdown, gifted students had a significantly lower motivation than non‐gifted students (a small effect), but during the lockdown, gifted and non‐gifted students did not differ significantly.

### Changes in perceived well‐being and motivation as a result of the lockdown and the role of need satisfaction in gifted and non‐gifted students

Four mediation models of MEMORE were run to examine to what extent a change in need satisfaction, because of the lockdown, affected changes in well‐being and motivation, separately in gifted students and non‐gifted students.

**Figure 2:**
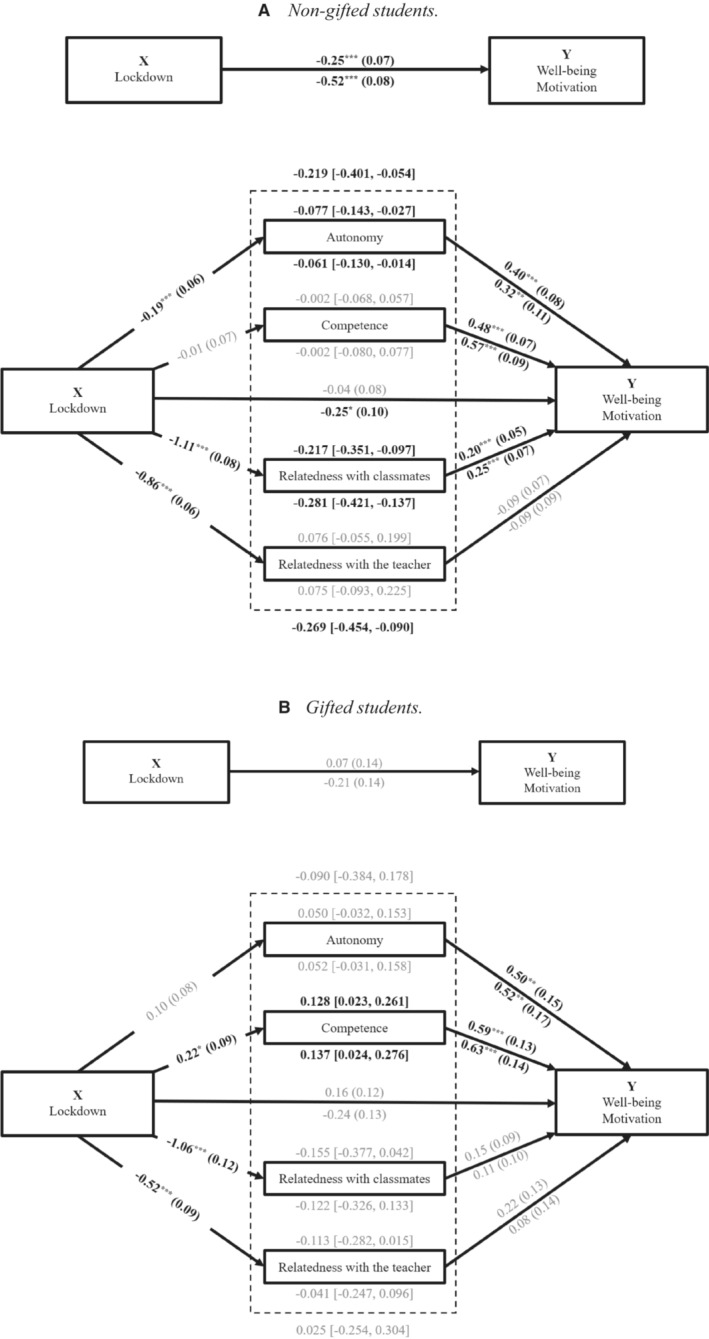
Four total‐effect and mediation models with lockdown as independent variable and well‐being and motivation as dependent variables, two models for non‐gifted students and two for gifted students. (a) Non‐gifted students. (b) Gifted students. *Note*. Above the arrows are the unstandardized coefficients (*B*) and standard errors (*SE*) of well‐being, and below the arrows are the results of motivation. Above the dashed box are the *B* and 95% confidence interval (CI) of the total indirect effect of well‐being, and below the dashed box the total indirect effect of motivation. Above the solid boxes of the needs are the *B* and the 95% CI of the indirect effects of well‐being, and below the solid boxes are the indirect effects of motivation. Black bold results are significant, and grey results are not significant. *P < 0.05, **P < 0.01, ***P < 0.001

Figure 2a shows the results of two mediation models for non‐gifted students. The lockdown had a negative total effect on non‐gifted students' well‐being and motivation: both decreased. The total indirect effect was significant: the effect of all mediators together. This total indirect effect was negative: In the non‐gifted students, satisfaction of the needs decreased due to the lockdown and as a result their well‐being and motivation decreased. However, when looking at the individual mediators, these decreases in well‐being and motivation were only significantly negatively mediated by a decrease in autonomy and relatedness with classmates’ satisfaction, not by a change in competence and relatedness with teacher satisfaction. Although non‐gifted students' relatedness with teacher satisfaction was much lower during than before the lockdown, this change did not affect changes in their well‐being and motivation. Finally, the lockdown had no direct effect on non‐gifted students' perceived well‐being and had a direct negative significant effect on their perceived motivation.

Figure 2b shows the results of two mediation models for gifted students. The total effects of the lockdown on well‐being and motivation were not significant, meaning that the lockdown did not affect gifted students' well‐being and motivation. The effect of all mediators together, the total indirect effect, was also not significant: A change in gifted students' satisfaction of the four needs, due to the lockdown, did not influence changes in their well‐being and motivation. However, when looking at the individual mediators, changes in gifted students' well‐being and motivation were significantly positively mediated by a change in satisfaction of the need for competence. For autonomy and relatedness satisfaction, we found no indirect effects in the gifted students, neither for well‐being nor for motivation. The lockdown had no direct significant effect on gifted students' perceived well‐being and motivation.

### Relationships between giftedness and perceived need satisfaction, well‐being and motivation before and during the lockdown

**Figure 3:**
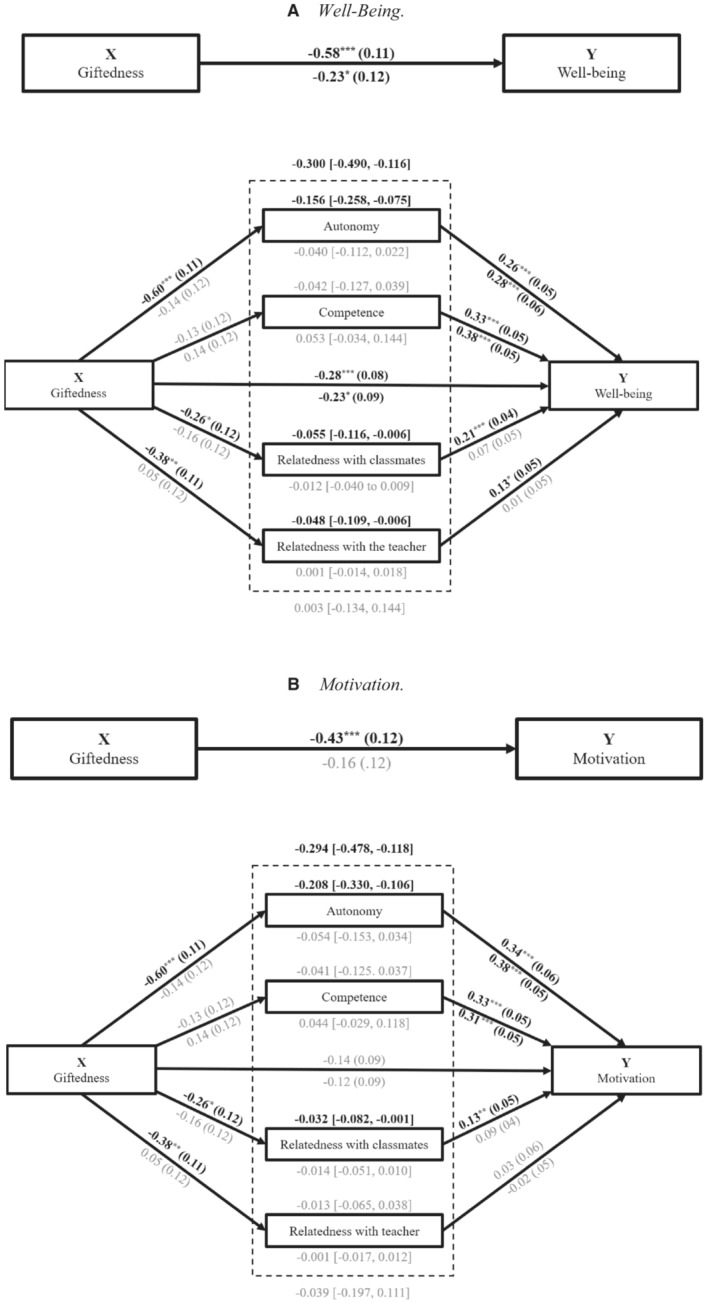
Four total‐effect and mediation models with giftedness as independent variable, two with well‐being as dependent variable and two with motivation as dependent variable. (a) Well‐being. (b) Motivation. *Note*. Above the arrows are the unstandardized coefficients (*B*) and standard errors (*SE*) before the lockdown, and below the arrows are the results during the lockdown. Above the dashed box are the *B* and 95% confidence interval (CI) of the total indirect effect before the lockdown, and below the dashed box the total indirect effect during the lockdown. Above the solid boxes of the needs are the *B* and the 95% CI of the indirect effects before the lockdown, and below the solid boxes are the indirect effects during the lockdown. Black bold results are significant, and grey results are not significant. *P < 0.05, **P < 0.01, ***P < 0.001

Four mediation models were run with PROCESS to investigate to what extent differences between gifted students and non‐gifted students in their well‐being and motivation were mediated by need satisfaction, see Figure 3.

Figure 3a presents the results of two mediation models with well‐being as dependent variable. Both before and during the lockdown, the total effect of giftedness on well‐being was significantly negative, meaning that giftedness is associated with lower well‐being. The total effect before the lockdown can be partly explained by a significant total indirect effect: the effect of all mediators together. This total indirect effect was negative: Gifted students generally had lower need satisfaction before the lockdown, leading to lower well‐being. When looking at the individual mediators, the relationship between giftedness and well‐being before the lockdown was negatively mediated by autonomy and relatedness satisfaction, but not by competence satisfaction. During the lockdown, none of the indirect effects was significant. Finally, the direct effect of giftedness on perceived well‐being was still negative and significant, both before and during the lockdown.

Figure 3b presents the results of two mediation models with motivation as dependent variable. Before the lockdown, giftedness had a negative total effect on motivation, meaning that before the lockdown, giftedness is associated with less motivation. During the lockdown, this effect was no longer significant. The total effect before the lockdown can be explained by a significant total indirect effect. This total indirect effect was negative: Lower need satisfaction of gifted students led to lower motivation before the lockdown. The relationship between giftedness and motivation before the lockdown was negatively mediated by autonomy and relatedness with classmates’ satisfaction, not by competence and relatedness with teacher satisfaction. During the lockdown, there was no significant indirect effect. Finally, giftedness had no direct effect on perceived motivation, neither before, nor during the lockdown.

## Discussion

The COVID‐19 lockdown and school closures affected children's lives in many different ways. We investigated the effects of the lockdown on the relationship between need satisfaction, well‐being and motivation for gifted and non‐gifted primary school students from a parental perspective. We expected that the effects of the lockdown were generally negative for all students, especially for non‐gifted students, and that a decrease in need satisfaction would explain decreases in well‐being and motivation. This hypothesis has been partly confirmed.

### Impact of the lockdown on students' perceived need satisfaction, well‐being and motivation

#### Non‐gifted students

As expected, the lockdown had a negative impact on non‐gifted students' need satisfaction, well‐being and motivation. Due to the lockdown, their *well‐being* and especially their *motivation* decreased. These decreases can be explained by a small decrease in their *autonomy* satisfaction and a large decrease in their *relatedness with classmates Hornstra* satisfaction. These results are consistent with the SDT, stating that a lower need satisfaction negatively affects well‐being and motivation (Church et al., 2013; Ryan and Deci, 2017). Autonomy may have decreased because students experienced less autonomy support and more parental control during the lockdown (Bülow et al., 2021; Stoecklin et al., 2021). In addition, their decrease in relatedness with classmates probably decreased because all contact with classmates was online. This finding is consistent with previous research, showing that social contacts decreased and students' feeling of isolation increased due to the lockdown (Bülow et al., 2021; Pozas et al., 2021; Thorell et al., 2020).

Contrary to our expectations, non‐gifted students' satisfaction of the need for *competence* did not decrease and this did not affect their well‐being and motivation. Probably, less guidance from schools during the lockdown (Thorell et al., 2020) was compensated by increased involvement of parents. Previous research showed that most parents were indeed involved in their children's online learning activities (Hafidz et al., 2020; Novianti and Garzia, 2020). Since our sample mainly consists of higher‐educated parents, it is likely that these parents were also able to guide and support their children with their homework, making their children feel competent. Other COVID‐19‐research showed indeed that higher‐educated parents supported their children more with their homework and felt more capable of doing so than lower‐educated parents (Bol, 2020). Finally, although the non‐gifted students felt much less related with their teacher during the lockdown, this did not affect their well‐being and motivation. It is plausible that an effect of *relatedness with teacher* was not found because students satisfied their need for relatedness with parents and siblings (Mangiavacchi et al., 2021; Pozas et al., 2021).

#### Gifted students

The impact of the lockdown was not the same for all students. In general, as expected, the impact was more negative for non‐gifted than for gifted students. The lockdown even had no overall effect on gifted students' *well‐being* and *motivation*. However, the influence of their need satisfaction was very variable. A very small increase in gifted students' competence satisfaction, as a result of the lockdown, predicted a small positive change in their well‐being and motivation. Although their *relatedness* satisfaction decreased strongly, their well‐being and motivation did not decrease because this was compensated by a slightly increased *competence* satisfaction. It is possible that their competence increased a little due to individual guidance of their parents (Hafidz et al., 2020; Novianti and Garzia, 2020), but also because they could make their schoolwork at their own pace. It was contrary to our expectations that the lockdown had no negative effect on gifted students' *autonomy* satisfaction. Possibly, parents provided equal levels of autonomy support as teachers, because of the special needs of their gifted child. A pre‐lockdown interview study showed that parents of gifted children indeed used more autonomy‐supporting than controlling strategies to stimulate their children's motivation for homework (Garn, Matthews and Jolly, 2010).

#### Gifted and non‐gifted students: A comparison

Because the lockdown did not have such a negative impact on gifted students as it did on non‐gifted students, the small‐to‐moderate differences between these groups decreased regarding their well‐being and motivation. Before the lockdown, gifted students had lower well‐being, motivation, autonomy satisfaction and relatedness with teacher satisfaction than non‐gifted students. During the lockdown, these differences became smaller for well‐being and even disappeared for motivation and autonomy and relatedness satisfaction, mainly driven by a (slightly stronger) decrease among the non‐gifted students. Only the small difference between gifted and non‐gifted students in their relatedness with classmates’ satisfaction remained about the same before and during the lockdown. Possibly gifted students, both before and during the lockdown, experienced less satisfaction of this need because they belong to a minority group. Being part of a minority group affects the way students are perceived and approached by others (Aboud et al., 2012). By being approached differently, gifted students may feel less related to their classmates.

Gifted students' lower well‐being and slightly lower motivation before the lockdown can be explained by less autonomy and relatedness with classmates’ satisfaction and for well‐being also by a lower relatedness with teacher satisfaction. Only this lower relatedness with teacher satisfaction was not in line with previous research (Hornstra et al., 2020). However, in that study, students themselves were questioned, while our study is from a parental perspective. Moreover, it was just a quite small difference in the present study. It is also possible that the relationship with teacher is slightly lower for gifted students because they often received less guidance from their teacher than other students and regular teaching is often not adjusted to their educational needs (Fraser‐Seeto et al., 2015). Anyhow, to reduce the differences between gifted and non‐gifted students in their well‐being and motivation, it is important to provide better support to gifted students to meet their needs of autonomy and relatedness. Since autonomy did not decrease in the gifted students, but it did decrease in the other group, gifted students can probably benefit more from an environment in which acting according to one's own will is supported.

Furthermore, it is remarkable that well‐being before and during the lockdown was not correlated. Students' well‐being changed in several ways: For some individuals, their well‐being decreased, for others their well‐being increased or it remained the same. It is therefore plausible that other individual and environmental factors may have influenced (changes in) gifted and non‐gifted students' well‐being.

### Limitations and suggestions for future research

A first limitation of the present study is that parental perspective was used, which can differ from the children's perspective (e.g., Sixsmith et al., 2007). Future (retrospective) studies could investigate the child's perspective in the older age group. A second limitation is that not all factors that could potentially moderate the effects of the lockdown on students' well‐being and motivation have been investigated (e.g., family factors). In future studies, a broader perspective including more factors related to circumstances could be included. Another limitation is that well‐being and motivation were measured by only one and two parallel items, while both are multidimensional constructs. Longer scales would probably have measured both constructs more adequately. Furthermore, a limitation is that gifted students were identified in different ways, which means that the group of gifted students may be very heterogeneous.

Our questionnaire was publicly available, allowing parents to decide for themselves whether or not to participate in our study. A disadvantage of this self‐selection may be that a specific group of parents participated in our study. Compared with the general population, our sample consisted of a relatively large number of highly educated parents and parents of gifted children, which could influence the results. However, the study by Hornstra et al. (2022) showed that the impact of the lockdown on need satisfaction, well‐being and motivation was generally not influenced by the educational level of parents. Nevertheless, our conclusions should be interpreted with caution, because the study by Hornstra and colleagues involved parents of secondary school students. These students may be less dependent on the support of their parents than primary school students. Although our sample cannot be directly generalized to the entire population and all educational contexts, the findings provide an impression of the impact of the lockdown on (gifted) students' need satisfaction, well‐being and motivation from a parental perspective. Moreover, our sample also had an advantage: The large response by parents of gifted children that this approach yielded enabled us to make a good comparison between gifted and non‐gifted students. For future research, schools could be approached to participate in research and ask the parents or students to participate. This might lead to a more representative sample. However, our approach enabled us to collect data quickly while it was uncertain how long the lockdown would last.

## Conclusion

The impact of the lockdown on primary school students' need satisfaction, well‐being and motivation was mainly negative. Students' well‐being and motivation decreased, mainly due to a decrease in autonomy and relatedness with classmates' satisfaction. However, the impact of the lockdown was less negative for gifted students. The lockdown had a levelling effect, as pre‐existing differences between gifted and non‐gifted students became smaller or even disappeared during the lockdown. This research emphasizes the importance for teachers to meet the needs of all students, including the gifted, and continuously assessing what else (gifted) students need for improved well‐being and motivation.

## Conflict of interest

The authors declare no conflict of interest.

## Ethics statement

Ethics approval for the study was granted by the institutional ethics committee of Fontys University.

## Data Availability

The research data are not shared due to privacy or ethical restrictions.
